# A multiplex RPA coupled with CRISPR-Cas12a system for rapid and cost-effective identification of carbapenem-resistant *Acinetobacter baumannii*

**DOI:** 10.3389/fmicb.2024.1359976

**Published:** 2024-03-06

**Authors:** Zihan Zhou, Lina Liang, Chuan Liao, Lele Pan, Chunfang Wang, Jiangmei Ma, Xueli Yi, Meiying Tan, Xuebin Li, Guijiang Wei

**Affiliations:** ^1^Center for Medical Laboratory Science, Affiliated Hospital of Youjiang Medical University for Nationalities, Baise, Guangxi, China; ^2^Baise Key Laboratory for Research and Development on Clinical Molecular Diagnosis for High-Incidence Diseases, Baise, Guangxi, China; ^3^Key Laboratory of Research on Clinical Molecular Diagnosis for High Incidence Diseases in Western Guangxi, Baise, Guangxi, China; ^4^Modern Industrial College of Biomedicine and Great Health, Youjiang Medical University for Nationalities, Baise, Guangxi, China

**Keywords:** *Acinetobacter baumannii*, recombinase polymerase amplification, CRISPR-Cas12a, nucleic acid detection, point-of-care testing

## Abstract

**Background:**

Carbapenem-resistant *Acinetobacter baumannii* (CRAB) poses a severe nosocomial threat, prompting a need for efficient detection methods. Traditional approaches, such as bacterial culture and PCR, are time-consuming and cumbersome. The CRISPR-based gene editing system offered a potential approach for point-of-care testing of CRAB.

**Methods:**

We integrated recombinase polymerase amplification (RPA) and CRISPR-Cas12a system to swiftly diagnose CRAB-associated genes, *OXA-51* and *OXA-23*. This multiplex RPA-CRISPR-Cas12a system eliminates bulky instruments, ensuring a simplified UV lamp-based outcome interpretation.

**Results:**

Operating at 37°C to 40°C, the entire process achieves CRAB diagnosis within 90 minutes. Detection limits for *OXA-51* and *OXA-23* genes are 1.3 × 10^−6^ ng/μL, exhibiting exclusive CRAB detection without cross-reactivity to common pathogens. Notably, the platform shows 100% concordance with PCR when testing 30 clinical *Acinetobacter baumannii* strains.

**Conclusion:**

In conclusion, our multiplex RPA coupled with the CRISPR-Cas12a system provides a fast and sensitive CRAB detection method, overcoming limitations of traditional approaches and holding promise for efficient point-of-care testing.

## 1 Introduction

*Acinetobacter baumannii* (*A. baumannii*), a Gram-negative pathogen, is widely distributed in the natural environment due to its rapid adaptability and low nutritional requirements (Pompilio et al., 2021). This characteristic allows it to persist in various hospital settings, imposing unnecessary burdens on the healthcare system in terms of diagnosis, logistics, and finances (Doughty et al., 2023). *A. baumannii* is a clinically significant nosocomial pathogen, and human infections with this bacterium can lead to complications such as meningitis (Fursova et al., 2023), bacteremia (Hong et al., 2023; Nutman et al., 2023) and ventilator-associated pneumonia (Riddles and Judge, 2023). Notably, *A. baumannii* is most commonly encountered in intensive care units (ICU) and critically ill patients, where infections contribute significantly to organ failure and sepsis, representing primary causes of patient mortality (Blanco et al., 2018; Seok et al., 2021). Carbapenems have historically served as frontline drugs in controlling *A. baumannii* infections. However, over the past few decades, widespread use of this class of drugs, coupled with *A. baumannii*’s intrinsic and acquired resistance traits, has led to the escalating prevalence of infections caused by Carbapenem-resistant *A. baumannii* (CRAB) (Raible et al., 2017). This resistance profile exhibits extensive antimicrobial resistance, further elevating its incidence and mortality rates (Hamidian and Nigro, 2019). Currently, only a limited number of drugs, such as polymyxins and tigecycline, are available for managing infections (Abdul-Mutakabbir et al., 2021). CRAB holds the designation of “Priority 1: CRITICAL” in the World Health Organization’s pathogen priority list, signifying a grave menace to global public health (Tacconelli et al., 2018). A survey conducted in Maryland involving 482 mechanically ventilated patients revealed a 30.7% infection rate with *A. baumannii*, with CRAB accounting for 88 cases (59.5%) (Harris et al., 2023). Additionally, an investigation reported similar CRAB loads in the environment surrounding almost all clinically or non-clinically CRAB-positive patients (Schechner et al., 2023). Hence, timely and effective strategies for the prevention and control of CRAB infections are crucial to limit their impact on hospital-acquired infections and mortality.

Presently, the predominant CRAB populations globally are classified into GC1 and GC2 clusters (Hamidian and Nigro, 2019). These populations exhibit resistance to carbapenem antibiotics through the production of various carbapenemases Such as KPC, IMP, NDM, VIM, etc. with the most common being class carbapenem-hydrolyzing class D oxacillinases (OXAs). Due to mutations on the OXAs, there are several different subgroups of the OXAs family, such as *OXA-51*, *OXA-23*, *OXA-24/40*, *OXA-48*, and *OXA-58* like. Endogenous *OXA-51* has a low level of carbapenemase expression and does not usually produce drug resistance. However, it is ubiquitous in *A. baumannii*, is highly species-specific, and widely recognized as a specific indicator for the identification of *A. baumannii* (Merkier and Centrón, 2006; Zander et al., 2012); *OXA-23* is the most frequently encountered carbapenemase gene and plays a key role in governing CRAB’s resistance to carbapenem drugs. Its prevalence has been extensively documented worldwide (Findlay et al., 2023; Huang et al., 2023). A report on the molecular epidemiology of CRAB from Chinese hospital indicated that the *OXA-51-like* gene was widely present in CRAB, while it was absent in all other isolates.The *OXA-23-like* gene was found in 97.7% of CRAB and was widely spread in our country (Wang et al., 2007).

Bacterial culture combined with antimicrobial susceptibility testing is still considered the gold standard for identifying CRAB (Yusuf et al., 2023). However, the extended turnaround time (2–3 days) and reliance on experienced technical personnel hinder the timely provision of rapid and accurate test reports to clinical settings. Polymerase chain reaction (PCR), has demonstrated significant advantages in the bacterial identification field due to its accuracy and speed, but standard PCR involves complex steps with large operational errors and is relatively time-consuming. Quantitative PCR (qPCR) relies on expensive and bulky real-time fluorescence amplification devices. The various limitations of these methods are not conducive to conducting rapid screening for CRAB in resource-limited areas. With the advancement of molecular diagnostics, isothermal amplification strategies have emerged as formidable alternatives, such as recombinase polymerase amplification (RPA), loop-mediated isothermal amplification (LAMP) (Wu et al., 2022), rolling circle amplification (RCA) (Deng et al., 2023) and multiple initiators and a rolling circle amplification (Sun et al., 2023). These technologies allow efficient amplification of target genes under isothermal conditions, eliminating the dependence on large equipment and facilitating the widespread adoption of molecular diagnostics. RPA offers a temperature-friendly reaction environment (37–42°C) and rapid response (15–25 min). Its potential for integration with point-of-care testing (POCT) makes it a promising candidate for replacing PCR in the future (James and Macdonald, 2015).

In some archaea, clustered regularly interspaced short polymorphic repeats (CRISPR) and CRISPR-associated proteins (Cas) form an adaptive immune system that plays an important role in defense against external infections in the natural environment (Raschmanová et al., 2018). The CRISPR-Cas system enables rapid and specific recognition of single nucleotides under isothermal conditions, significantly impacting the field of biosensing. It has emerged as a potent tool in nucleic acid detection, particularly in advancing POCT (Chen et al., 2023; Zhao et al., 2023). Proteins such as Cas12a, Cas12b, and Cas13a, renowned for their robust collateral cleavage activity, find widespread applications in the detection of viruses, bacteria, and single-nucleotide polymorphisms (Gootenberg et al., 2017; Chen et al., 2018). In contrast to the CRISPR-Cas13a system that targets RNA, the Cas12a protein, guided by crRNA, forms a crRNA-Cas12a-target ternary complex with the target DNA. This induces a conformational change in Cas12a, leading to specific cleavage of the target chain DNA and non-specific cleavage of surrounding single-stranded DNA (ssDNA) reporters. Coupled with other sensing strategies, Such as fluorescence detection or lateral flow strips. This property facilitates straightforward visual detection. Leveraging these features, the CRISPR-Cas12a system is particularly well-suited for bacterial detection and has rapidly advanced, demonstrating successful applications in detecting common pathogens such as *Escherichia coli* (Zhu et al., 2023), *Pseudomonas aeruginosa* (Liu et al., 2023), and *Salmonella typhi* (Duan et al., 2024).

Utilizing the multiplex RPA and CRISPR-Cas12a system, we devised a visual readout platform targeting the endogenous gene *OXA-51* and the carbapenem-resistant gene *OXA-23* in CRAB. This platform leverages RPA to amplify the *OXA-51* and *OXA-23* genes within 25 min. Subsequently, the amplicons are introduced into the CRISPR-Cas12a system, which specifically recognizes *OXA-51* and *OXA-23*, respectively. Results can be visualized using a UV lamp within 30 min.

## 2 Materials and methods

### 2.1 Reagents and apparatus

CRAB standard strain was procured from Biobw Co., Ltd. (Beijing, China). Standard strains of *A. baumannii* (ATCC 19606), *Escherichia coli* (ATCC 25922), *Staphylococcus aureus* (ATCC 25923), *Klebsiella pneumoniae* (ATCC 700603), *Pseudomonas aeruginosa* (ATCC 27853), and *Stenotrophomonas maltophilia* (ATCC 19861) were provided by our laboratory strain bank. Clinical strains of carbapenem-resistant *K. pneumoniae* (CRKP) and carbapenem-resistant *P. aeruginosa* (CRPA) were isolated and identified by our microbiology laboratory and genomic extracts were performed by molecular biology laboratory. The source of the strains and their resistance to carbapenems are shown in Table 1. 30 clinical isolates of *A. baumannii* and 20 non-*A. baumannii* were provided by clinical laboratory for the purpose of evaluating clinical efficacy. All clinical strains were identified using the VITEK® 2 Compact microbial identification system (Lyon, French).

**Table 1:** Source of bacterial strains used in this study and carbapenem resistance.

Bacteria	Source	Sensitivity for carbapenems (P/N)
*A. baumannii*	ATCC 19606	P
*E. coli*	ATCC 25922	P
*S. aureus*	ATCC 25923	P
*K. pneumoniae*	ATCC 700603	P
*P. aeruginosa*	ATCC 27853	P
*S. maltophilia*	ATCC 19861	N
CRAB	Biobw Co., Ltd	N
CRKP	Clinical isolation	N
CRPA	Clinical isolation	N

CRAB, carbapenem-resistant *Acinetobacter baumannii*; CRKP, carbapenem-resistant *Klebsiella pneumoniae*; CRPA, Carbapenem-resistant *Pseudomonas aeruginosa*. P, positive; N, negative.

The Twist AmpTM Basic Kit for RPA was purchased from Twist DX Co., Ltd. (Hertfordshire, UK). EnGen® Lba Cas12a (Cpf1) and NEBuffer r2.1 were obtained from New England Biolabs (Guangzhou, China). The 2× Es Taq MasterMix (Dye) for PCR was purchased from Cwbio Co., Ltd. (Jiangsu, China). Fluorescence readings were recorded using the TIANLONG Gentier 96E/96R qPCR analysis system (Xi’an, China). All PAGE-purified oligonucleotides, including RPA and PCR primers, crRNAs, and ssDNA, were ordered from Sangon Biotech Co., Ltd. (Shanghai, China).

### 2.2 Bacterial culture and genomic DNA extraction

The seven standard strains utilized in this study were maintained in 20% glycerol and stored at −80°C for prolonged preservation. Genomic DNA extraction followed the TIANamp Bacteria DNA Kit guidelines. The concentration of genomic DNA was assessed using a spectrophotometer, with a qualified sample defined by an OD260/280 value ranging between 1.8 and 2.0.

For extracting genomic DNA from the clinical strains, a boiling method was employed: The strains were gathered in EP tubes, washed twice with PBS, centrifuged at 13,000 g for 2 min, and the supernatant was discarded. Subsequently, TE buffer (100 μL) was introduced, followed by boiling for 10 min. Afterward, centrifugation at 13,000 g for 5 min was conducted to collect the supernatant. All DNA templates were stored at −20°C before use in experiments.

### 2.3 Primers and crRNAs design

The gene sequences for *OXA-51* (GenBank: KP462889.1) and *OXA-23* (GenBank: KF740470.1), targeting CRAB, were obtained from NCBI.^1^ Using the NCBI online primer design tool “Primer-BLAST” and following the instructions of the Twist AmpTM Basic Kit, four primer pairs were designed for each gene and validated for specificity. Specific parameters were set as follows: Primer pair length between 28–35 bp, GC content between 30–70%, amplicon length between 150–300 bp, Tm value between 50–100%, and a maximum allowable length of a mononucleotide repeat setting of “5.”

Crucial for the specificity of CRISPR-Cas system recognition, crRNA plays a key role, and low-activity crRNA can lead to unpredictable errors in the CRISPR-Cas system (Javaid and Choi, 2021; Park et al., 2023). Therefore, we searched for the position of the protospacer adjacent motif (PAM, TTTN) in the amplicons of *OXA-51* and *OXA-23* (Supplementary Figure S1). Three crRNAs were designed for each amplicon to select the two most efficient ones. The detailed information on primers, crRNAs, and ssDNA reporter involved in this study was summarized in Supplementary Table S1.

### 2.4 Standard RPA

Standard RPA is used for primer pair screening. In the 50 μL RPA amplification system, following the Twist AmpTM Basic Kit instructions, 29.5 μL of rehydration buffer was added to the lyophilized enzyme powder (containing recombinase, polymerase, and single-stranded binding protein, etc.), and slight shaking was performed to dissolve it. Subsequently, 2.4 μL each of forward and reverse primers (10 μM) were added to create the amplification premix. Finally, 4 μL of the template, 2.5 μL of MgOAc, and ddH_2_O were added to make up the volume to 50 μL, Mixing with shaking and centrifugation.

In order to reduce costs and improve the utilization of reagents, a 10 μL amplification system was established, including 8.7 μL of the premix, 0.8 μL of DNA template, and 0.5 μL of MgOAc. The reaction proceeded at 37°C for 20 min. Amplification products were detected using 2% agarose gel electrophoresis.

### 2.5 CRISPR-Cas12a cleavage

For the amplification of *OXA-51* and *OXA-23*, two sets of fluorescence detection systems were designed. The 20 μL cleavage system consisted of 2 μL NEBuffer r2.1 (10×), 2 μL amplification product, 500 nM fluorescence reporter (ssDNA, 5’FAM-TTATT-BQ1’3), 50 nM LbCas12a (1 μM), and 50 nM crRNA (*OXA-51*/*OXA-23*). After amplification by multiplex RPA, the amplified products were separately added to the two systems. The cleavage process was monitored using a qPCR instrument: The reaction proceeded at 37°C for 30 min, and fluorescence intensity was recorded every 60 s. The generated fluorescence signals were analyzed using GraphPad Prism 9 (GraphPad Software, United States), and fluorescence was excited under UV light, with images captured using a smartphone.

### 2.6 Optimization of multiplex RPA-CRISPR-Cas12a system

To realize the amplification of *OXA-51* and *OXA-23*, a multiplex RPA amplification system was established on a standard RPA, and the reaction parameters were optimized and adjusted, including primer concentration ratios, reaction time, and temperature. To mitigate potential competitive inhibition resulting from multiple primer pairs, concentration gradients ranging from 240 nM to 560 nM were individually tested for the two primer sets to determine the optimal ratio. Subsequently, a range of amplification temperatures (34°C, 37°C, 40°C, and 43°C) and amplification times (20 min, 25 min, 30 min, and 35 min) were systematically explored to identify the optimal reaction conditions. The outcomes were assessed via 2% agarose gel electrophoresis.

Furthermore, to ensure the efficiency of the non-specific cleavage in CRISPR-Cas12a systems, optimization was performed for the concentration of Cas12a and crRNA. To keep the initial system constant, Cas12a concentrations of 50 nM, 100 nM, 150 nM, 200 nM, and 250 nM were tested. After determining the optimal Cas12a concentration, the concentration of crRNA was varied (100 nM, 150 nM, 200 nM, 250 nM) and the best combination was selected based on fluorescence intensity. Negative control was blank RPA amplification and fluorescence readings were recorded by qPCR instrument. Each experiment was repeated three times.

### 2.7 Sensitivity and specificity of multiplex RPA-CRISPR-Cas12a system

To investigate the sensitivity of the multiplex RPA-CRISPR-Cas12a system, ddH_2_O was used as a diluent, and CRAB DNA genomic was diluted in tenfold gradients from 1.3 × 10^−2^ ng/μL to 1.3 × 10^−6^ ng/μL as templates. The optimized multiplex RPA-CRISPR-Cas12a system was employed for validation and compared with multiplex RPA and PCR. Subsequently, we verified the specificity of the system by detecting the genomics of other clinically common pathogens, including standardized *A. baumannii* (no *OXA-23*), *E. coli, S. aureus*, *K. pneumoniae*, *P. aeruginosa*, *S. maltophilia*, CRKP and CRPA. Each experiment was repeated three times.

### 2.8 Validation of clinical strains

To demonstrate the utility of the multiplex RPA-CRISPR-Cas12a system, 30 clinical strains suspected of *A. baumannii* were collected from the clinical laboratory, with 26 strains (84%) originating from the ICU. In addition, 20 strains of non-*A. baumannii* were included in the study. Genomic DNA was extracted using a boiling method followed by assaying and comparing the consistency of the two methods using the multiplex RPA-CRISPR-Cas12a system and PCR kits. The 25 μL PCR system including 12.5 μL of 2× Es Tap MasterMix (Dye), 0.4 μM each of forward and reverse primers, 2 μL template, and finally the volume was replenished to 25 μL with ddH_2_O. The PCR cycling program consisted of an initial denaturation at 94°C for 2 min, followed by 35 cycles of denaturation at 94°C for 30 s, annealing at 53°C for 30 s, extension at 72°C for 30 s, and a final extension at 72°C for 2 min. Subsequently, the amplification products underwent analysis through 2% agarose gel electrophoresis.

## 3 Results and discussion

### 3.1 Workflow of the multiplex RPA-CRISPR-Cas12a detection system for CRAB

**Figure 1:**
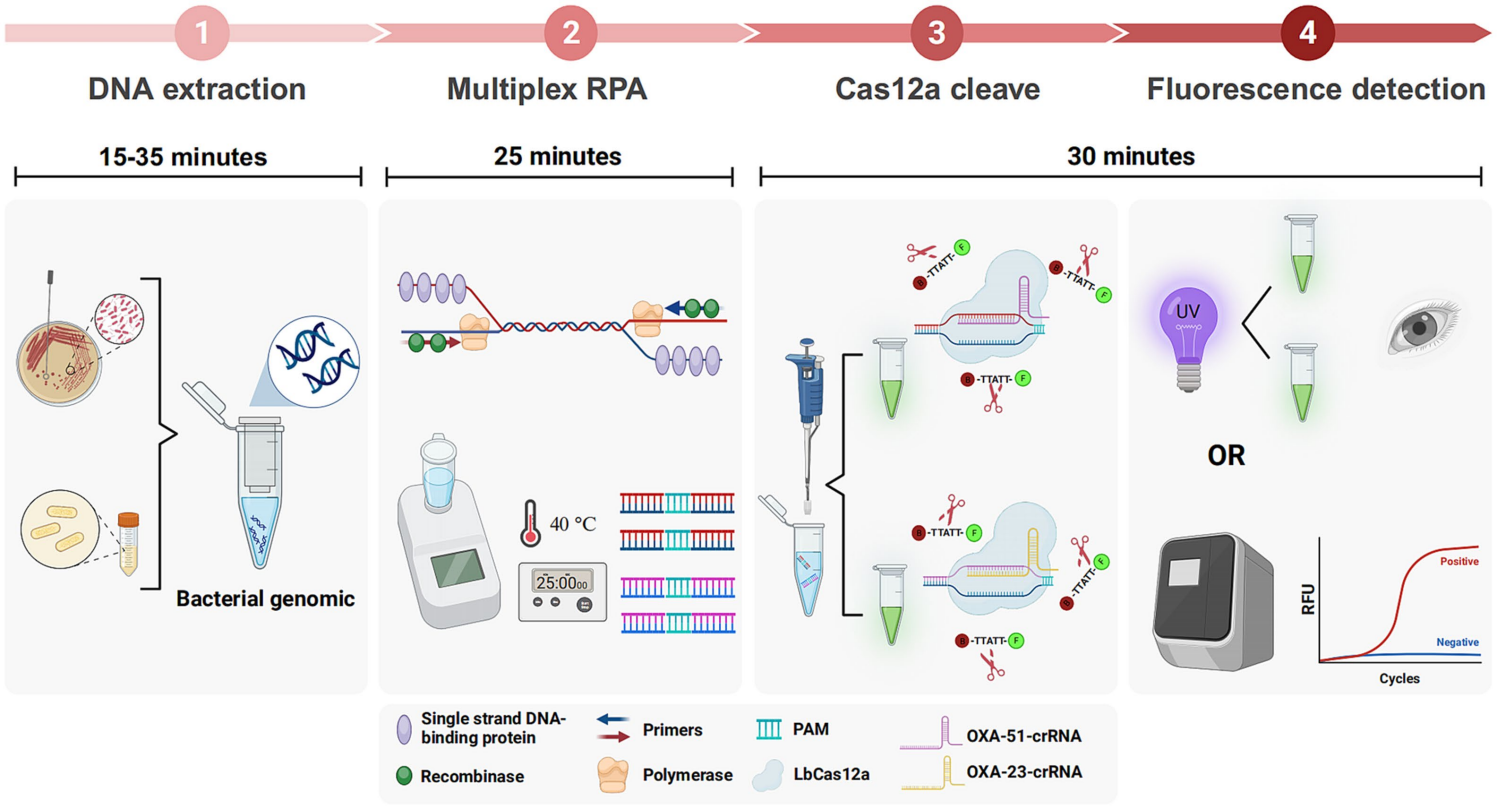
Workflow of multiplex RPA-CRISPR-Cas12a system. Multiplex RPA was used for exponential amplification of *OXA-51* and *OXA-23*. The amplification products were subsequently added to the CRISPR-Cas12a system that can specifically recognize *OXA-51* and *OXA-23*, respectively. Finally, the results can be interpreted by a real-time fluorescence amplifier or UV light.

The schematic in Figure 1 elucidates the CRAB detection process integrating multiplex RPA with CRISPR-Cas12a. Genomic DNA extraction from samples, using either a commercial kit or a simplified boiling method, precedes RPA amplification of two target fragments. This rapid amplification, completed within approximately 25 min, is driven by essential components such as recombinase, single-strand binding protein, and primers. The amplified products are then introduced into the CRISPR-Cas12a system, designed for *OXA-51* and *OXA-23* amplicons.

The crRNA-Cas12a complex showcases specificity by recognizing the PAM site on double-stranded DNA, forming a binding with the target strands via complementary base pairing. Cas12a undergoes a conformational change, leading to cleavage of the target strands and indiscriminate cleavage of the surrounding ssDNA reporter. Results are visually discernible under ultraviolet or blue light, offering a straightforward and rapid detection method. The entire procedure can be completed in less than 90 min.

### 3.2 Screening of RPA primers

**Figure 2:**
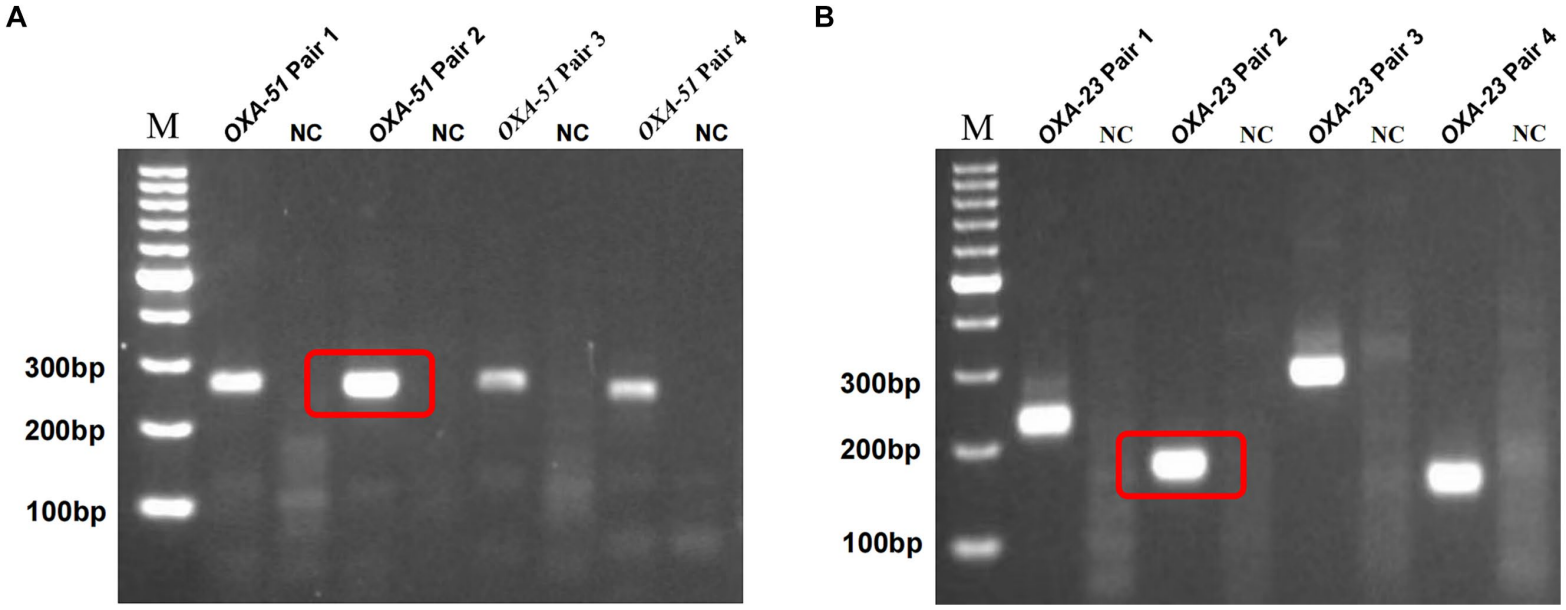
Screening of multiplex RPA primers. M, marker. NC, negative target control. *OXA-51* Pair 2 **(A)** and *OXA-23* Pair 2 **(B)** showed the brightest bands without hairpin structures such as dimers, and the negative control showed no non-specific amplification.

Building on prior research, *OXA-51* and *OXA-23* stand out as recognized endogenous genes of *A. baumannii* and carbapenemase-encoding genes. For each gene, we designed four primer pairs, with detailed sequences and product lengths available in Supplementary Table S1. The optimal primer pairs were meticulously chosen via standard RPA reactions, followed by 2% agarose gel electrophoresis, revealing successful amplification of target segments in all cases. Figures 2A,B show the results of screening by gel electrophoresis, showcasing that all primer pairs effectively amplified the target segments. Notably, *OXA-51* Pair 2 and *OXA-23* Pair 2 demonstrated prominent bands devoid of dimers or hairpin structures. Consequently, these two primer pairs were selected for subsequent experiments.

### 3.3 Optimization conditions for multiplex RPA-CRISPR-Cas12a system

Expanding on the standard RPA, we developed a multiplex RPA system and fine-tuned its conditions. Optimization efforts focused on the concentration ratio of the two primer sets, reaction time, and temperature. Optimal amplification efficiency for both primer sets was achieved at 400 nM for *OXA-23* and 320 nM for *OXA-51* (Supplementary Figure S2). Subsequent tests involved varying amplification times (15, 20, 25, and 30 min) and reaction temperatures (34°C, 37°C, 40°C, and 43°C). The highest amplification efficiency surfaced at 25 min and a reaction temperature of 40°C.

**Figure 3:**
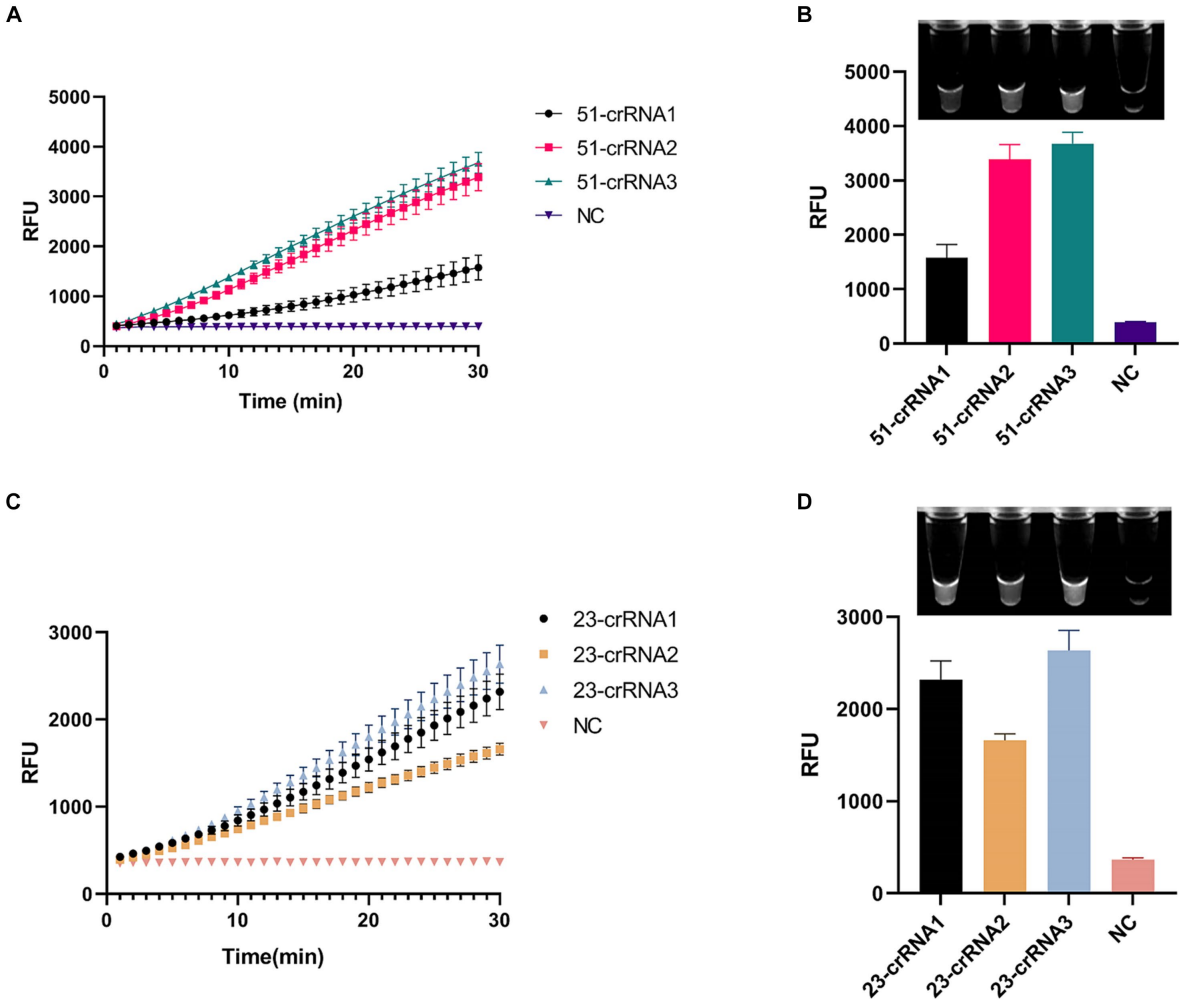
Screening of crRNA of multiplex RPA-CRISPR-Cas12a system. NC, negative target control. **(A,B)** show the fluorescence curves and endpoint signals of three crRNAs designed for the *OXA-51* amplicon. **(C,D)** show the fluorescence curves and endpoint signals of three crRNAs designed for the *OXA-23* amplicon. *N* = 3, error bars indicated the standard deviation.

**Figure 4:**
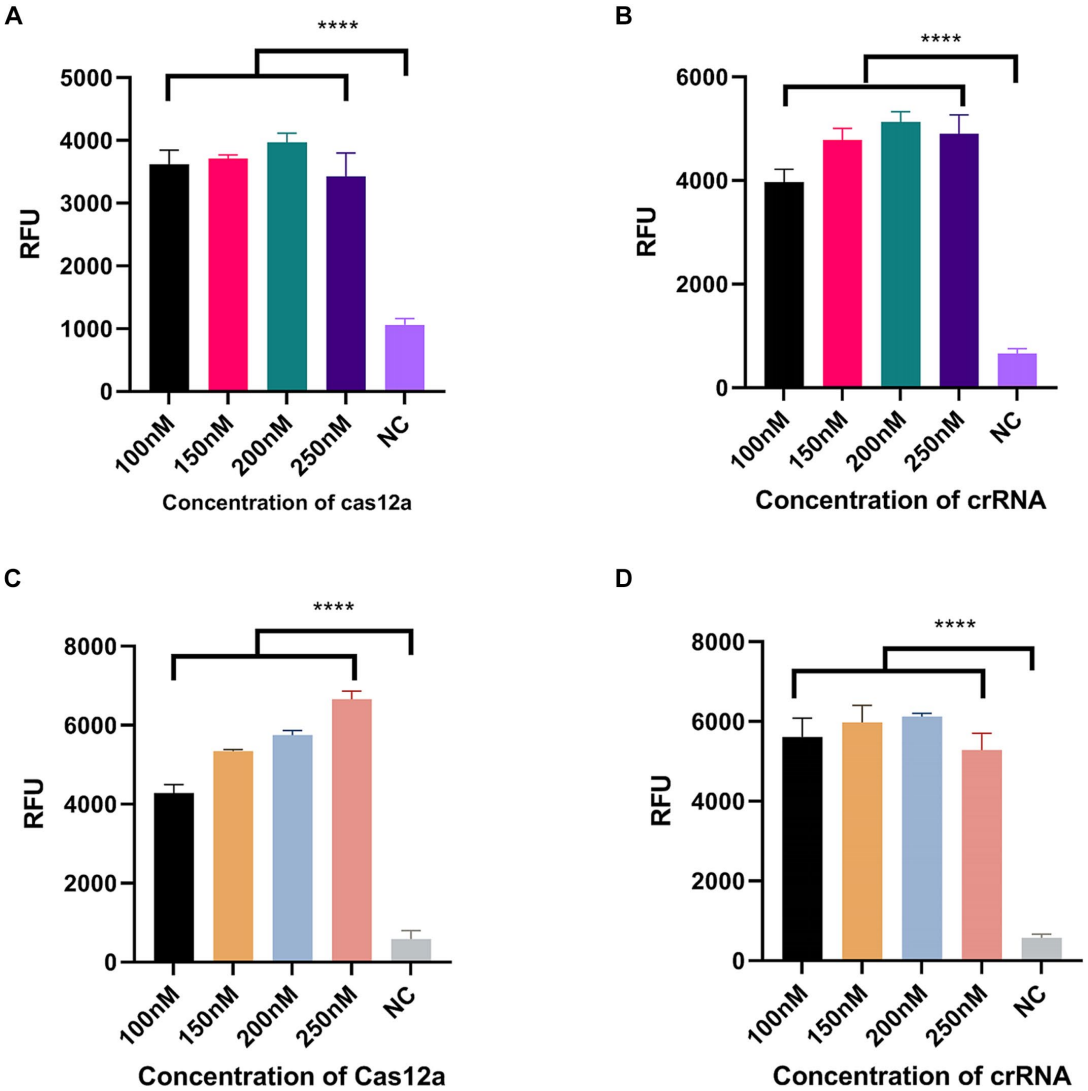
Optimization of CRISPR-Cas12a system component. NC, negative target control. **(A,B)** shows the Cas12a concentration and crRNA concentration of the CRISPR-Cas12a detection system for *OXA-51*. **(C,D)** shows the Cas12a concentration and crRNA concentration of the CRISPR-Cas12a detection system for *OXA-23. N* = 3, error bars indicated the standard deviation. Two-tailed Student’s *t*-test; ^****^p < 0.0001.

We designed three crRNAs for *OXA-51* and *OXA-23* amplicons, respectively, to screen out the most efficient one. Each crRNA was successful in directing the specific recognition of Cas12a, but only 51-crRNA3 (Figures 3A,B) and 23-crRNA3 (Figures 3C,D) showed the highest non-specific cleavage efficiency within 30 min. Finally, optimization of the reaction components in the CRISPR-Cas12a system was performed. As depicted in the optimization program, fluorescence curves indicate that for the CRISPR-Cas12a detection system targeting *OXA-51*, the highest fluorescence efficiency was achieved when Cas12a concentration was 200 nM and crRNA concentration was 150 nM (Figures 4A,B). In the case of the *OXA-23* detection system, a significant fluorescence value was observed when the Cas12a concentration was 250 nM. Due to cost considerations, further optimization was not pursued. An optimal fluorescence efficiency was also obtained with crRNA concentration at 200 nM (Figures 4C,D).

### 3.4 Sensitivity and specificity of multiplex RPA-CRISPR Cas12a

**Figure 5:**
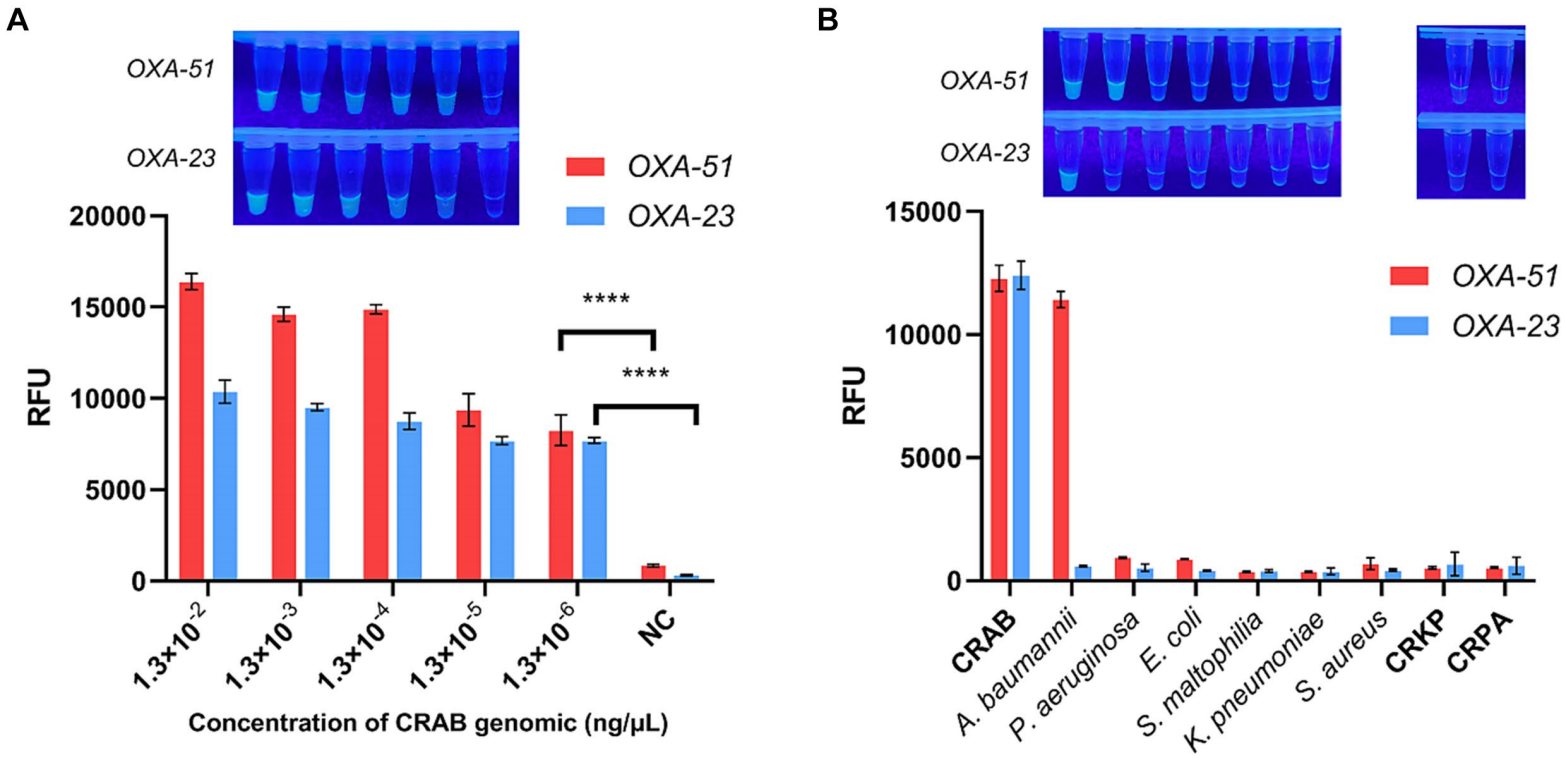
Sensitivity and specificity of multiplex RPA-CRISPR-Cas12a. NC, negative target control. **(A)** Fold dilution of the CRAB genomic DNA was used to validate the sensitivity of multiplex RPA-CRISPR-Cas12a. **(B)** Validation of multiplex RPA-CRISPR-Cas12a specificity using six strains of pathogenic bacteria commonly found in the clinic. *N* = 3, error bars indicated the standard deviation. Two-tailed Student’s *t*-test; ^****^p < 0.0001.

The sensitivity of the multiplex RPA-CRISPR-Cas12a system was assessed through a gradient dilution of CRAB genomic DNA ranging from 1.3 × 10^−2^ ng/μL to 1.3 × 10^−6^ ng/μL. As shown in Figure 5A, the endpoint fluorescence showed that the fluorescence signal could still be detected when the genomic concentration was 1.3 × 10^−6^ ng/μL, demonstrating a two-order-of-magnitude improvement compared to multiplex RPA and and a one-order-of-magnitude improvement compared to PCR (Supplementary Figure S3). This suggests that multiplex RPA-CRISPR-Cas12a is a potentially more sensitive tool for CRAB detection.

To confirm the specificity of the multiplex RPA-CRISPR-Cas12a system, tests were conducted on clinically common pathogenic bacteria, including *E. coli*, *S. aureus*, *K. pneumoniae*, *P. aeruginosa*, *S. maltophilia*, CRKP and CRPA. It was found that only CRAB and *A. baumannii* displayed a fluorescent signal, indicating no cross-reactivity with other strains, affirming the system’s specificity (Figure 5B).

### 3.5 Detection of clinical strains by multiplex RPA-Cas12a system

**Figure 6:**
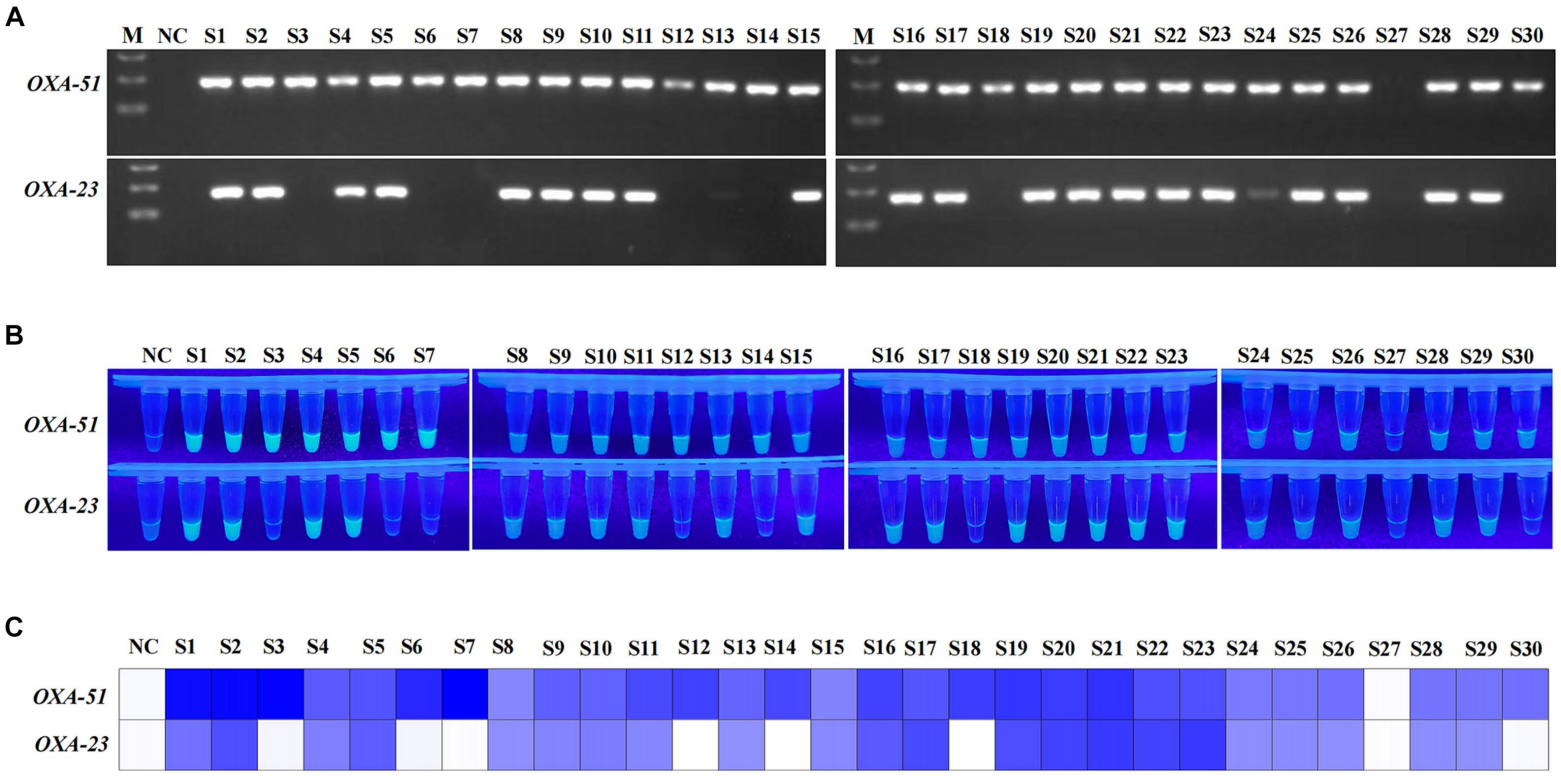
Detection of clinical strains of *A. baumannii*. NC, negative target control. S1-S30, *A. baumannii* obtain from clinical. M, Marker. **(A)** Agarose gel image showing the clinical strains tested with the PCR assay. **(B,C)** show the visualised signals and the heatmap of endpoint fluorescence values of 30 clinical strains detected using multiplex RPA-CRISPR-Cas12a.

The clinical performance of the multiplex RPA-Cas12a system was evaluated by conducting simultaneous PCR and multiplex RPA-CRISPR-Cas12a analyses on all 30 *A. baumannii* and 20 of non-*A. baumannii*. PCR results revealed amplification of the *OXA-51* gene in 29 clinical strains (29/30), confirming them as *A. baumannii*. Among them, 22 strains were concurrently detected with the *OXA-23* gene (22/29), identifying them as CRAB, while 1 sample was negative (Figure 6A). 20 non-*A. baumannii* clinical strains did not show amplified bands and were determined to be negative(The results of non-*A. baumannii* test were not presented). Subsequent results from the multiplex RPA-CRISPR-Cas12a system were 100% consistent with the PCR method by using UV reads (Figures 6B,C). The facts of the above study indicate that the system we developed is comparable to PCR and is a reliable tool for the detection of CRAB (Table 2).

**Table 2:** Multiplex RPA-CRISPR Cas12a and PCR for clinical strains detection.

multiplex RPA-CRISPR-Cas12a	PCR			Total
	CRAB	AB	Negative	
CRAB	22	0	0	22
AB	0	7	0	7
Negative	0	0	21	21
Total	22	7	21	50

CRAB, Carbapenem-resistant *A. baumannii*; AB, *A. baumannii*.

### 3.6 Discussion

CRAB, a notorious drug-resistant strain, poses a significant threat to hospital infection control, especially in developing countries where its prevalence and mortality rates categorize it as a serious public health concern. CRAB commonly resides in patients’ respiratory, skin, urinary, and reproductive tracts, causing a variety of symptoms. Upon breaching the circulatory system, it triggers severe bloodstream infections and can invade the cranial cavity, leading to conditions such as sepsis and meningitis. Its acquired resistance characteristics have led to the identification of clinically pan-resistant strains, fostering widespread outbreaks in ICU. According to China’s 2021 CHINT data, *A.baumannii* ranks second among pathogens isolated from the respiratory tract, exhibiting resistance rates of 65.5 and 66.5% to imipenem and meropenem, respectively. This scenario presents substantial challenges to clinical diagnosis and treatment. However, the lack of data on the prevalence of CRAB infections in low-income countries is a major obstacle, primarily due to the absence of monitoring tools capable of timely detection of such infections (Rizk et al., 2021). Therefore, there is an urgent need for a rapid and sensitive detection method to facilitate early prevention, detection, and treatment of CRAB in public environments and vulnerable populations.

Commonly used clonality identifications associated with *A. baumannii* are usually based on multilocus sequence typing of multiple genes, and although highly accurate, these methods are time-consuming and expensive. In contrast, a single-gene assay based on *OXA-51* or *OXA-23* appears to be a cheaper and more convenient method of detection (Zander et al., 2012). CRISPR, as a revolutionary gene-editing system, has been widely applied in molecular diagnostics research in recent years. It offers advantages such as simplicity in synthesis, ease of use, and high specificity. Initially, the crRNA, serving as a guide strand, forms an effector complex with Cas protein. Upon recognizing the target sequence upstream with a sequence adjacent to the “PAM” rich in thymine (T), the cutting activity of the effector is activated. While it specifically cuts the target strand, it also non-specifically cuts the surrounding ssDNA. However, relying solely on the CRISPR system for molecular diagnostics often proves inefficient, particularly when dealing with low concentrations and the presence of inhibitors (Gootenberg et al., 2017; Wang et al., 2021). In contrast, RPA is a widely-used isothermal amplification method, stands out for its rapidity, sensitivity, and a certain tolerance to inhibitors. Integrating the RPA amplification with the specific recognition capabilities of the CRISPR-Cas system markedly enhances both sensitivity and specificity in detection. This integrated approach has gained prominence as a preferred POCT strategy in recent years. Additionally, other isothermal amplification technologies such as LAMP (Yang et al., 2023) and RCA (Wang et al., 2020) can also be coupled with the CRISPR-Cas system to meet the diverse detection requirements of different targets.

In this study, we developed a platform integrating multiplex RPA with the CRISPR-Cas12a fluorescence detection system, enabling rapid diagnosis of CRAB in both laboratory and POCT. The platform achieves swift diagnosis of CRAB’s dual genes within 90 min, encompassing sample processing, amplification, Cas12a cleavage, and result interpretation. The required equipment includes only a water bath, a mini-centrifuge, an isothermal heating module, and a UV or blue light source. By reducing the volume of the multiplex RPA to one-fifth of the recommended volume, the cost of the test is further compressed, making it more accessible to hospitals in low-resource areas, with testing costs as low as $2 per sample. Under open laboratory conditions, the sensitivity of the method is at least 1.3 × 10^−6^ ng/μL, showing an improvement in detection limit by at least two orders of magnitude compared to RPA and PCR. Moreover, in the validation with clinical samples, it achieved 100% concordance with the PCR method.

Our study was based on the *OXA-51* and *OXA-23* genes. Despite the prevalence of both genes in most of the CRAB, there are still slip-ups in clinical trials (Figure 6; S27). The possible reason is that the strain belongs to a small number of carbapenemase subspecies, which need to be further characterized by relying on a multigene multiplex assay. The repetitive opening and transferring steps inevitably lead to aerosol contamination, causing false-positive issues, particularly in open laboratory conditions. Additionally, the single-tube CRISPR-Cas12a can only detect one gene, and the dual-tube approach increases reagent costs. Current research is exploring the integration of multiplex RPA with Cas12a/Cas13a, developing a one-pot detection method (Tian et al., 2022). However, this compromised one-pot approach achieves the spatial separation of target amplification and CRISPR signal amplification by physically separating them. For instance, effective separation can be achieved by utilizing glycerol to separate RPA and CRISPR phases or by directly attaching CRISPR to the tube wall (Tian et al., 2022; Kang et al., 2023). While these methods to some extent reduce the contamination risks associated with multiple lid-opening operations, they still require additional manual centrifugation steps. Furthermore, the reduced reaction volume in one-pot detection may compromise sensitivity and introduce operational errors (Hu et al., 2020; Xiao et al., 2023). In conclusion, the universality of the current one-pot strategy for RPA-CRISPR still awaits further verification. In the future, we will continue to optimize our strategy to minimize excessive manipulations and move towards a more integrated single-tube operation.

## 4 Conclusion

In summary, our study presents a CRISPR-Cas12a detection system utilizing multiplex RPA, offering notable advantages in terms of speed, sensitivity, and ease of use. This platform facilitates the swift and simultaneous identification of the *OXA-51* and *OXA-23* genes in CRAB. Its applicability for early and rapid CRAB infection diagnosis holds promise in curbing their potential for extensive spread, particularly within hospital environments. Furthermore, this system presents an economically efficient screening approach tailored for regions with limited resources.

## Data availability statement

The datasets presented in this study can be found in online repositories. The names of the repository/repositories and accession number(s) can be found in the article/Supplementary material.

## Ethics statement

Written informed consent was obtained from the individual(s) for the publication of any potentially identifiable images or data included in this article.

## Author contributions

ZZ: Conceptualization, Investigation, Validation, Visualization, Writing – original draft. LL: Conceptualization, Writing – original draft. CL: Data curation, Writing – review & editing. LP: Data curation, Writing – review & editing. CW: Resources, Validation, Writing – review & editing. JM: Resources, Validation, Writing – review & editing. XY: Resources, Validation, Writing – review & editing. MT: Data curation, Writing – review & editing. XL: Conceptualization, Writing – review & editing. GW: Conceptualization, Supervision, Writing – review & editing.

## Footnotes

^1^https://www.ncbi.nlm.nih.gov/
