# Supplementary material for: A multiplex RPA coupled with CRISPR-Cas12a system for rapid and cost-effective identification of carbapenem-resistant Acinetobacter baumannii
Source: Front Microbiol. 2024 Mar 6;15:1359976. doi: 10.3389/fmicb.2024.1359976 (PMC10956356; doi:10.3389/fmicb.2024.1359976)
Supplement: Supplementary file 1 [file Data_Sheet_1.docx]

Supplementary Material

| **Name** | **Sequence (5'-3')** | **Amplicon Length(bp)** |
| --- | --- | --- |
| RPA-OXA-51-F1 | CATAAGGCAACCACCACAGAAGTATTTAAG | 255 |
| RPA-OXA-51-R1 | CTCTTGCTGAGGAGTAATTTTTAAAGGA |  |
| RPA-OXA-51-F2 | TAAGGCAACCACCACAGAAGTATTTAAG | 254 |
| RPA-OXA-51-R2 | CCTCTTGCTGAGGAGTAATTTTTAAAGG |  |
| RPA-OXA-51-F3 | CATAAGGCAACCACCACAGAAGTATTTA | 240 |
| RPA-OXA-51-R3 | CCTCTTGCTGAGGAGTAATTTTTAAAGGA |  |
| RPA-OXA-51-F4 | CCATAAGGCAACCACCACAGAAGTATTTA | 256 |
| RPA-OXA-51-R4 | ATTTTTAAAGGACCCACCAGCCAAAAATTA |  |
| RPA-OXA-23-F1 | CGCGCAAATACAGAATATGTGCCAGCCTCT | 217 |
| RPA-OXA-23-R1 | GATCAAGACCGATACGTCGCGCAAGTTCCT |  |
| RPA-OXA-23-F2 | GCCCTGATCGGATTGGAGAACCAGAAAACG | 170 |
| RPA-OXA-23-R2 | AGATCAAGACCGATACGTCGCGCAAGTTCC |  |
| RPA-OXA-23-F3 | GCGCGACGTATCGGTCTTGATCTCATGCAA | 294 |
| RPA-OXA-23-R3 | GCCGGTCAACCAGCCCACTTGTGGTTTTAT |  |
| RPA-OXA-23-F4 | GCCCTGATCGGATTGGAGAACCAGAAAACGG | 157 |
| RPA-OXA-23-R4 | TACGTCGCGCAAGTTCCTGATAGACTGGGA |  |
| PCR-OXA-51-F | AGCACCATAAGGCAACCA | 181 |
| PCR-OXA-51-R | CCAACACGCTTCACTTCC |  |
| PCR-OXA-23-F | TGATCGGATTGGAGAACCAG | 166 |
| PCR-OXA-23-R | AGATCAAGACCGATACGTCG |  |
| ssDNA | FAM-TTATT-BHQ1 | / |
| 51-crRNA1 | UAAUUUCUACUAAGUGUAGAUAUGGCAUCGCCUAGGGUCAU | / |
| 51-crRNA2 | UAAUUUCUACUAAGUGUAGAUUCAAGAUUUAGCUCGUCGUA | / |
| 51-crRNA3 | UAAUUUCUACUAAGUGUAGAUGCUCGUCGUAUUGGACUUGA | / |
| 23-crRNA1 | UAAUUUCUACUAAGUGUAGAUAAUGGAAGGGCGAGAAAAGG | / |
| 23-crRNA2 | UAAUUUCUACUAAGUGUAGAUCCAAGCGGUAAAUGACCUUU | / |
| 23-crRNA3 | UAAUUUCUACUAAGUGUAGAUCCGCUUGGGAAAAAGACAUG | / |

**Supplementary Table 1**. Sequence of primers, crRNAs and ssDNA.


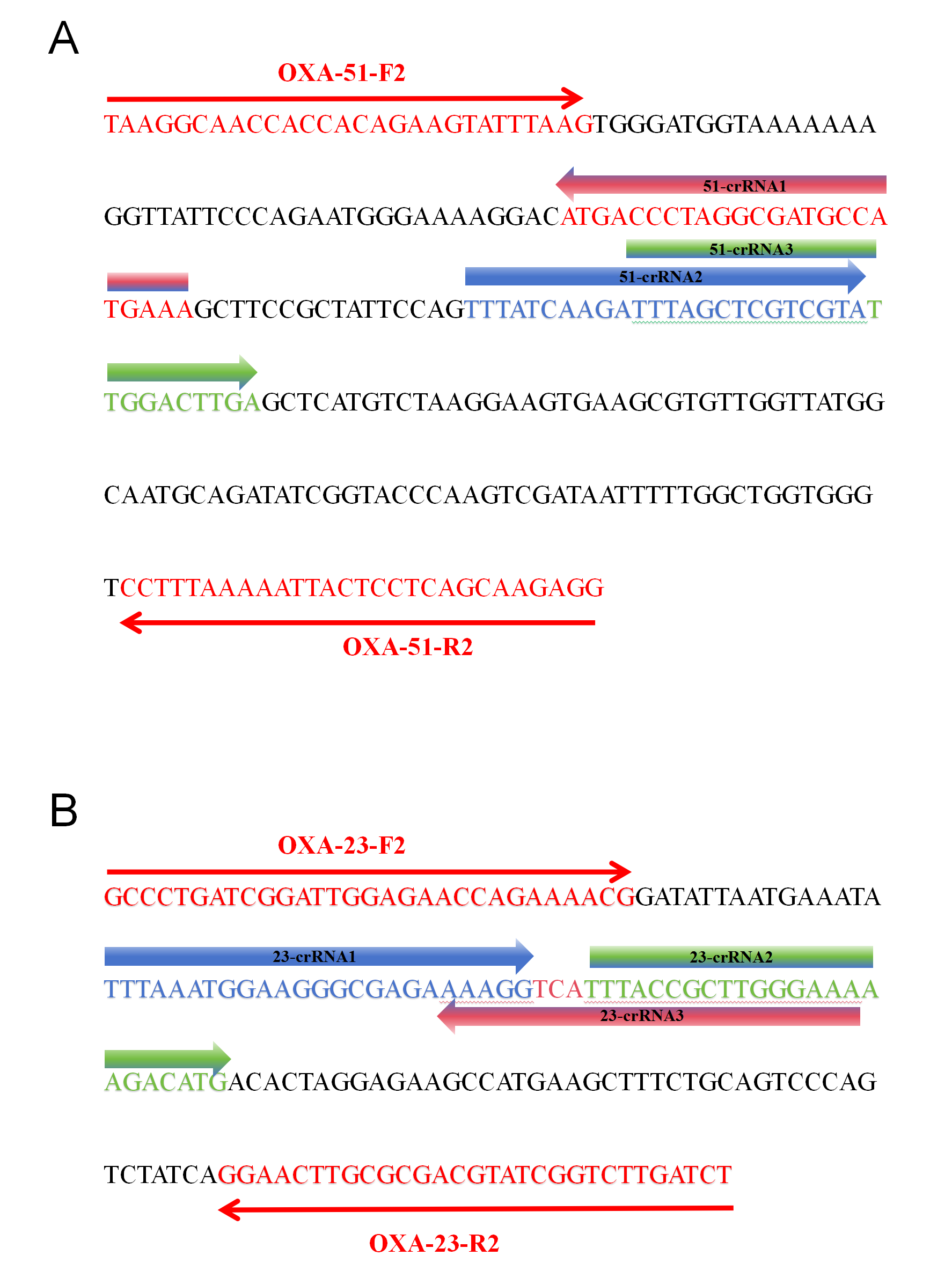


**Supplementary Figure S1.** Sequence and position of RPA primers and crRNAs on *OXA-51* (A) and *OXA-23* (B) amplicons. The long red arrows indicate the corresponding primer pairs.

**
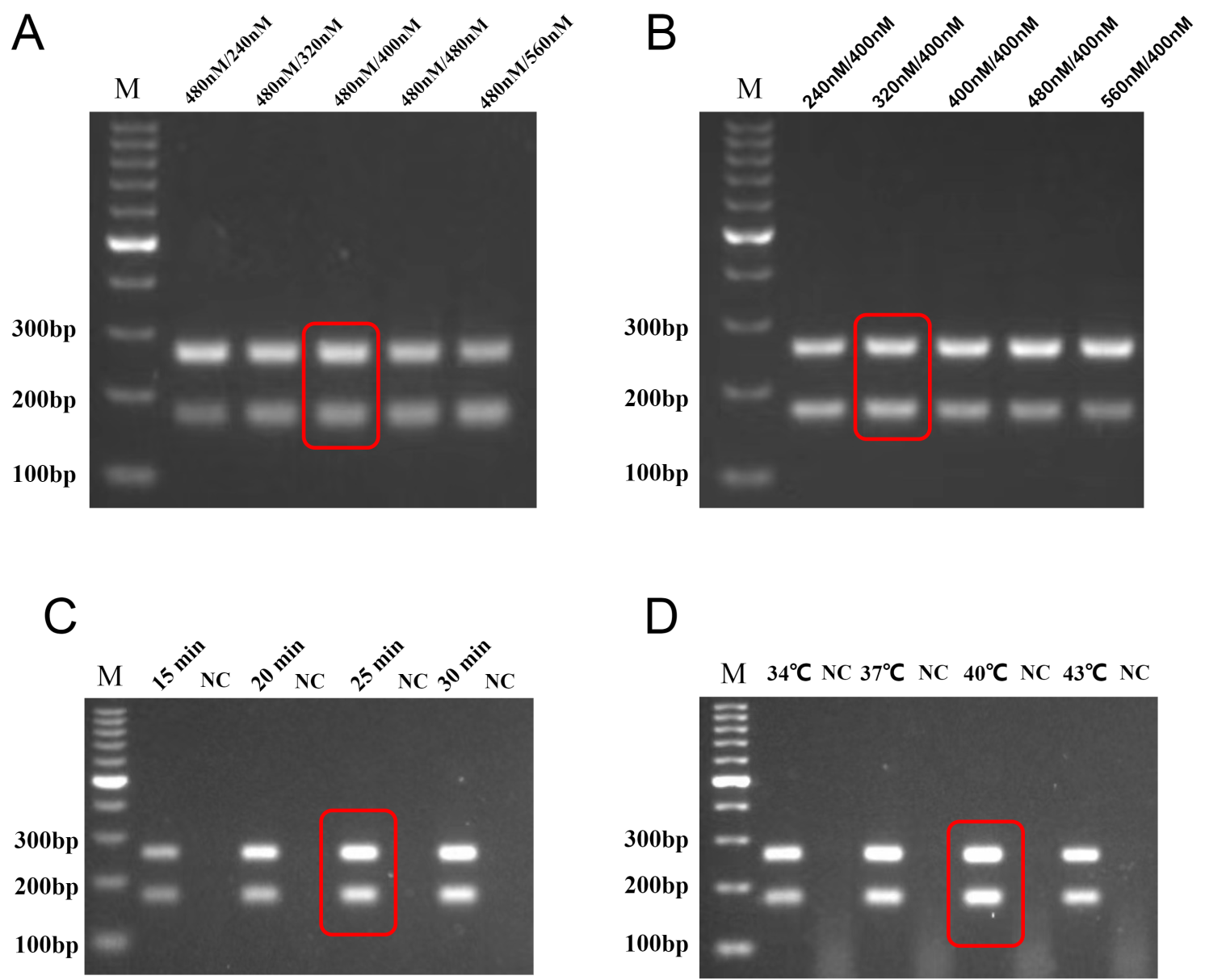
**

**Supplementary Figure S2.** Optimization of multiplex RPA reaction conditions. M, marker. NC, negative target control. (A) Optimization of primer concentrations for the *OXA-23* gene. (B) Optimization of primer concentrations for the *OXA-51* gene. (C) Optimization of amplification time. (D) Optimization of amplification temperature.


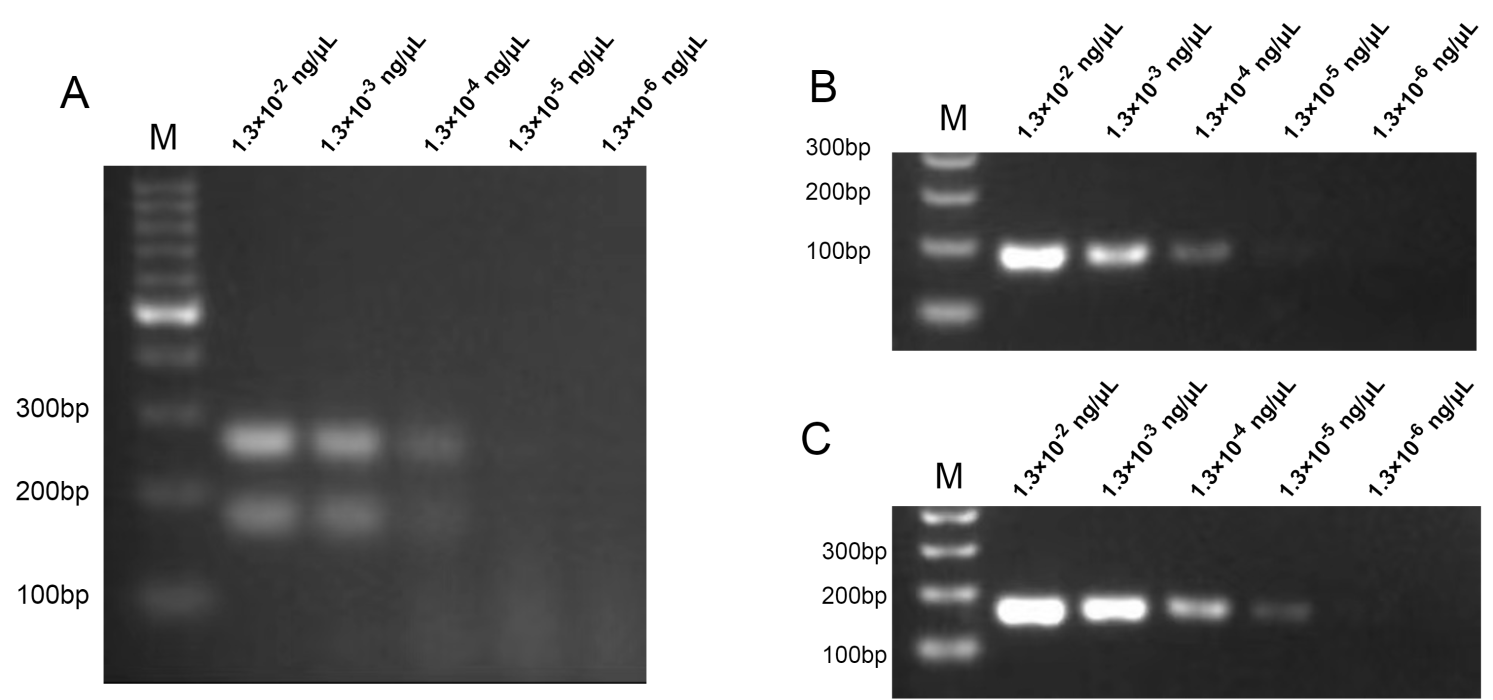


**Supplementary Figure S3.** Sensitivity of Multiplex RPA and PCR. M, marker. NC, negative target control. (A) The limit of detection for multiplex RPA can reach 1.3 × 10^-4^ ng/μL. (B) and (C) The limit of detection for PCR can reach 1.3 × 10^-5^ ng/μL.
